# Appetitive Aggression as a Resilience Factor against Trauma Disorders: Appetitive Aggression and PTSD in German World War II Veterans

**DOI:** 10.1371/journal.pone.0050891

**Published:** 2012-12-12

**Authors:** Roland Weierstall, Sina Huth, Jasmin Knecht, Corina Nandi, Thomas Elbert

**Affiliations:** Department of Psychology, University of Konstanz, Konstanz, Germany; University of California, San Francisco, United States of America

## Abstract

**Background:**

Repeated exposure to traumatic stressors such as combat results in chronic symptoms of PTSD. However, previous findings suggest that former soldiers who report combat-related aggression to be appetitive are more resilient to develop PTSD. Appetitive Aggression should therefore prevent widespread mental suffering in perpetrators of severe atrocities even after decades.

**Methods and Findings:**

To test the long-term relationship between trauma-related illness and attraction to aggression, we surveyed a sample of 51 German male World-War II veterans (age: *M = *86.7, *SD = *2.8). War-related appetitive aggression was assessed with the Appetitive Aggression Scale (AAS). Current- and lifetime PTSD symptoms were assessed with the PSS-I. In a linear regression analysis accounting for 31% of the variance we found that veterans that score higher on the AAS show lower PSS-I symptom severity scores across their whole post-war lifetime (*β = *− .31, *p* = .014). The effect size and power were sufficient (*f* ^2^ = 0.51, (1-*β*) = .99). The same was true for current PTSD (*β = *− .27, *p* = .030).

**Conclusions:**

Appetitive Aggression appears to be a resilience factor for negative long-term effects of combat experiences in perpetrators of violence. This result has practical relevance for preventing trauma-related mental suffering in Peace Corps and for designing adequate homecoming reception for veterans.

## Introduction

With over 60 million killed, the Second World War was the deadliest military conflict in history. Half of the human losses were soldiers [1]. In Germany alone, some 6 million people lost their lives while over 16 million were displaced, homeless or threatened by starvation [2].

Research in conflict regions it has demonstrated that civilian victims of war have a significantly increased risk for the development of serious trauma related mental health problems like posttraumatic stress disorder (PTSD), anxiety disorders or depression (e.g. [3]). The significant prevalence rates of PTSD in war affected populations result from the multitude of traumatic events people in conflict zones are exposed to: The greater the number of traumatic events experienced, the greater the risk for the development of trauma spectrum disorders. Due to the deleterious effects of cumulative exposure to traumatic stress on mental health (also termed “building block effect”; [4]), nearly 100% of people that have experienced two dozen or more traumatic event types suffer from PTSD [5]. However, the higher prevalence's of PTSD are not limited to victims of war but also may affect those who participate in combats and perpetrated the violence. Research on traumatic stress conducted with soldiers from World War II, prior to the introduction of PTSD into DSM, indicated symptoms of hyperarousal and anxiety in those who returned from the front line which had been termed traumatic war neurosis or shell shock [6], [7]. While the early research of ex-combatants and later the US-Vietnam veterans in particular, shaped the concept of PTSD, effects of active participation in combat haven't been studied systematically. The National Vietnam Veteran Readjustment Study (NVVRS) has shown a close relationship between the exposure to war-related traumatic events contributed and the development of PTSD in veterans [8], [9]. Even though attempts were made to define selection criteria to sort out those who are vulnerable for traumatisation [10], trauma spectrum disorders are still common in soldiers who have fought in current armed conflicts like in Iraq or Afghanistan [11] PTSD may become a chronic condition, persisting for decades or even for the rest of the life (e.g. [12], [13]). Even decades after the end of World War II studies with American World War II veterans reported PTSD rates of 16 to 29% and revealed that the effects of trauma have sustained [14], [15], [16], [17].

Armed conflicts and the eliminations of the out-group are common features of war and have always accompanied human history [18], [19], [20]. In these conflicts, soldiers are often actively engaged in fighting and killing. During evolution of man, hunting behaviour has evolved from vegetarian ancestors. Moreover, those who went hunting not only animals, but also killed members of the out-group were probably favoured by evolutionary selection. Consequently, such behaviour must have become appetitive and rewarding by itself [20], [21] irrespectively of subsequent gains like resources [22] or status [23]. We define ‘appetitive aggression’ as the perpetration of violence and/or the infliction of harm on a victim for the purpose of experiencing violence-related enjoyment. A human drive towards cruel behaviour can therefore be highly functional [24], [25]. It also seems to protect the hunter against trauma-related illness. In recent studies with Rwandan genocide perpetrators and with Ugandan child soldiers we could demonstrate that those who reported a greater propensity to appetitive aggression were more resilient towards the development of PTSD [26], [27]. Therefore, appetitive aggression not only achieves gains over status and power, but also prevents perpetrators from getting traumatized in the course of their own atrocities and it allows adaptation to a cruel environment. We presume that uncontrolled expression of the innate appetitive aggression is inhibited by frontal cortical mechanisms that are shaped during the process of socialization. Under circumstances like those in war, when moral barriers erode and violence is legitimated, cruel behavior and appetitive aggression can break through and foster an escalation of mass violence [28], [29].

The aim of the present study was to investigate whether the adaptive advantage in combatants who experience aggression to be appetitive also had very long-term protective and maybe even beneficial effects on their mental health. We surveyed a sample of male German World War II veterans. According to our results from former studies on combatants we predicted that those who report violence during war to be appetitive would be less prone to developing war related PTSD provided that the number of experienced traumatic event types is controlled for. Appetitive Aggression was assessed with the German version of the Appetitive Aggression Scale [30], [31].

## Methods

### Ethics Statement

The Ethical Review board of the University of Konstanz approved the study and all participants gave their written informed consent.

### Subjects

We obtained data from 51 World War II male Veterans that all experienced the Second World War as Germans. Table 1 gives an overview of the demographic data. Information about the military background of the Veterans is listed in Table 2. The convenience sample was recruited in different cities in the South-West of Germany by placing advertisements in the local newspapers and posting signs on bulletin boards in residential homes for the elderly as well as places for senior citizens to meet. Data was collected between May and September 2010 using structured interviews. All interviews were carried out in participants' homes and lasted for about 90 minutes. Three clinical psychologists from the University of Konstanz that were experts in the assessment of PTSD and trauma spectrum disorders as well as human aggression performed the interviews. The Mini-Mental Status Test (MMST [32]) was administered to all participants for the assessment of global cognitive impairment. The score indicating dementia was set at 24 of 30 or less. None of the participants met this dementia criterion. All participants gave written informed consent. They received no financial compensation.

**Table 1:** Demographic Data (Male veterans, n = 51).

**Age**, mean (*SD*), [range]	86.7 years (2.8) [81–95]
**Education**, No. (%)	
No school, some primary school	4 (7.9%)
Primary school	35 (49.0%)
Secondary School	6 (11.8%)
University-entrance diploma	16 (31.4%)
**Marital status**, No. (%)	
single	2 (3.9%)
married	31 (60.8%)
partner/cohabiting	1 (2.0%)
divorced	1 (2.0%)
widowed	16 (31.4%)
**No. Children**, mean (*SD*), [range]	2.2 (1.4) [0–5]
**No. grandchildren**, mean (*SD*), [range]	3.1 (3.2) [0–12]

**Table 2:** Military Data (male veterans, n = 51).

**Military draft age**, mean (*SD*), [range]	18.2 (1.5) [15–22]
**Conscription**, No. (%)	
induction	40 (78.4%)
voluntary	9 (17.6%)
other	1 (2.0%)
**Duration of military training**	
(month), mean (*SD*), [range]	5.6 (4.5) [1–24]
**Duration of combat mission**	
(month), mean (*SD*), [range]	26.8 (19.0) [1–70]
**Unit**, No. (%)	
army	38 (74.5%)
airforce	6 (11.8%)
marine	3 (5.9%)
other	2 (3.9%)
**Reasons for joining the military**	
mean (*SD*), [range 0–4]	
force	2.9 (1.5)
financial benefit	0.3 (0.9)
honour	0.8 (1.2)
hoping for adventure	0.7 (1.3)
military romanticism (comradeship, field exercises)	1.1 (1.3)
committed to the goals of war	1.2 (1.5)
serve the fatherland	1.8 (1.6)

Information about the military background of the Veterans is listed in Table 2. Moreover, we assessed seven different reasons for joining the military forces. Participants had to rate on a 5-point Likert scale to which extend the presented reasons were relevant for them, ranging from “0” (not at all) to “4” (“extremely”). The results are given in Table 2 too.

### Instruments

#### Assessment of traumatic experiences and PTSD symptoms

We assessed current as well as lifetime PTSD with the PSS-I [33]. The PSS-I assesses the severity of each of the 17 DSM-IV PTSD symptoms. The PSS-I has high test–retest reliability (*r* = 0.80) and inter-rater reliability (*k* = 0.91). For current PTSD the occurrence of PTSD symptoms in the period of the past 2 weeks was assessed (*PTSD symptom severity current*). For lifetime PTSD we asked for a period of at least two weeks in which the participants experienced PTSD symptoms most severe (*PTSD symptom severity lifetime*). Each symptom is rated on a 4-point scale from 0 (not at all) to 3 (very much). A sum score was calculated for each PTSD measure. The assessment of PTSD symptoms in structured interviews has been successfully validated in different populations of veterans [34], [35], [36]. The exposure to traumatic events was investigated using a modified PSS-I event scale. The original event scale was extended using war related items like violent house searches, abduction, displacements or life threatening hunger. Events were only rated as traumatic when they fulfilled the “A” criterion according to DSM IV, i.e. objective life-threat as well as subjective experience of intense fear, helplessness, or horror. We assessed whether the traumatic events were experienced during or outside wartime. For the further analysis, we also distinguished between the 18 items that dealt with self-experienced traumatic events and 16 items that dealt with witnessed event types. A traumatic event type was judged as *self-experienced* if the participant was the victim and as *witnessed* if the participant has seen or heard the traumatic event while someone else's life was threatened. We thus calculated four traumatic event scores for self-experienced and witnessed event types as well as events during and outside wartime.

#### Anxiety and depression symptoms

The associated symptoms of depression and anxiety were investigated using the Hopkins Symptom Checklist (HSCL [37]). The HSCL depression scale consists of 15 items whereas the anxiety scale contains 10 items. Coefficient Alpha in our sample was .84 for the anxiety scale and .77 for the depression scale. For each symptom, the participants have to rate on a 4-point Likert scale, how much they were bothered by the symptoms during the last two weeks. Both scales reflect the symptoms in correspondence with ICD criteria. We used the M.I.N.I. suicidality module to quantify current suicidality risk.

#### Appetitive Aggression

The participants' propensity towards violence was investigated with the German Version of the Appetitve Aggression Scale [30]. This 15-item questionnaire has been validated with more than 2.000 data sets among others from different war affected regions (e.g. Uganda, Congo, Rwanda and Columbia) showing a satisfactorily high reliability (Coefficient Alpha of .85) and validity. In this questionnaire statements regarding the perception of violence have to be rated as either true or not true by the participant. After probing each statement, responses to all items are rated on a 5-point Likert scale ranging from 0 (“I totally disagree”) to 4 (“I totally agree”). A sum score is calculated from all responses. The items are based on the subtype of instrumentally used aggression according to Vitiello and Stoff [38], the ICD 10 criteria for addiction and on perpetrators' self reports collected during the work in war affected crises regions (e.g. “Is it exciting for you if you make an opponent really suffer?”, “Is defeating the opponent more fun for you, when you see them bleed?”, “Once you got used to being cruel, did you want to be crueller and crueller?”). In line with the general assumption of a dichotomy of aggression, we have proven that appetitive aggression, as one facet of instrumentally used aggression, is different from reactive aggression [31].

### Data analysis

Linear regression analysis was used to demonstrate the impact of the number of traumatic events as well as a person's appetitive violence experience on the PTSD symptom severity. Correlations between variables were calculated using Spearman correlation coefficient. The statistical analysis was carried out with SPSS 20.0 and R for Apple Version 2.11.1.

## Results

### Trauma exposure, PTSD and associated psychiatric disorders

All participants reported war related items as worst events. 7.8% of the male veterans met the criteria for lifetime PTSD, but one of the veterans fulfilled the complete DSM IV criteria for a current PTSD diagnosis. According to the building block effect (increasing likelihood of trauma-related illness with increasing exposure) we regressed lifetime PTSD symptom severity on the number of traumatic event types experienced during as well as outside wartime. We further distinguished between the number of self-experienced and the number of witnessed traumatic event types. For both, current as well as lifetime PTSD symptom severity, the number of self-experienced traumatic event types during war was the only statistically significant predictor (lifetime PTSD symptom severity: *β = *.49, *p*<.001; current PTSD symptom severity: *β = *.48, *p*<.001). We further calculated Pearson correlation coefficients between the lifetime and current PTSD symptom severity scores with the HSCL anxiety and depression score (Table 3). Participants that reported a higher severity of current PTSD symptoms were also more burdened with co-morbid psychiatric disorders. Moreover, suffering from PTSD symptoms during lifetime in the aftermath of the World War also affected the current mental health status. As indicated by the correlation between lifetime and current PTSD symptom severity, those who developed trauma related distress during their life also currently experienced symptoms of trauma spectrum disorders.

**Table 3:** Intercorrelations between current and lifetime PTSD severity scores with depression, anxiety and suicidality scores.

	PTSD lifetime	HSCL	HSCL
		depression	anxiety
Current PTSD symptom			
severity score	.844	.243	.289
(*M* = 5.33; *SD* = 6.22)	*p*<.001	*p*<.080	*p* = .ß36
Lifetime PTSD symptom			
severity score	*-*	.372	.378
(*M* = 9.78; *SD* = 9.70)		*p* = .009	*p* = .008
HSCL depression score		-	.409
(*M* = 4.84; *SD* = 4.79)			*p* = .002
HSCL anxiety score			-
(*M* = 3.19; *SD* = 4.12)			

### Appetitive Aggression and its relation to trauma sypmtomatology

The appetitive aggression scale score ranged from 0 to 31 points (*M = *6.*0*, *SD = *6.2). To test our hypothesis, we performed two linear forced-entry regression analyses to analyse the factors contributing to current as well as lifetime PTSD symptom severity. We regressed lifetime PTSD symptom severity measured by the PSS-I score on the number of traumatic event types experienced during wartime as well as the appetitive aggression score in both models. Only the traumatic event types experienced during wartime were used, as the previous analysis has revealed that only these event types were a strong predictor for trauma-related symptoms.

In line with our hypothesis and previous results, the appetitive aggression score was a negative predictor for lifetime PTSD symptom severity (*β = *− .31, *p* = .014), i.e. veterans who have experienced the exposure to violence during war as more appealing were also less likely to develop PTSD symptoms in the aftermath (*F*_2_ = 11.55, *p*<.001, *R* ^2^_adj_ = .31), while the building block effect indicated by the relation between the number of self-experienced event types and the PSS-I score has remained (*β = *.52, *p*<.001). The same model was also applicable for the prediction of the current PTSD symptom severity (appetitive aggression score: *β = *− .27, *p* = .030; self-experienced event types: *β = *.50, *p*<.001; model summary: *F*_2_ = 10.50, *p*<.001, *R* ^2^_adj_ = .27). The interaction terms of self-experienced event types and the appetitive aggression scale scores did neither reach statistical significance nor did they improve the Akaike Information Criterion (AIC; [39]) as a measure for model fit.

**Figure 1:**
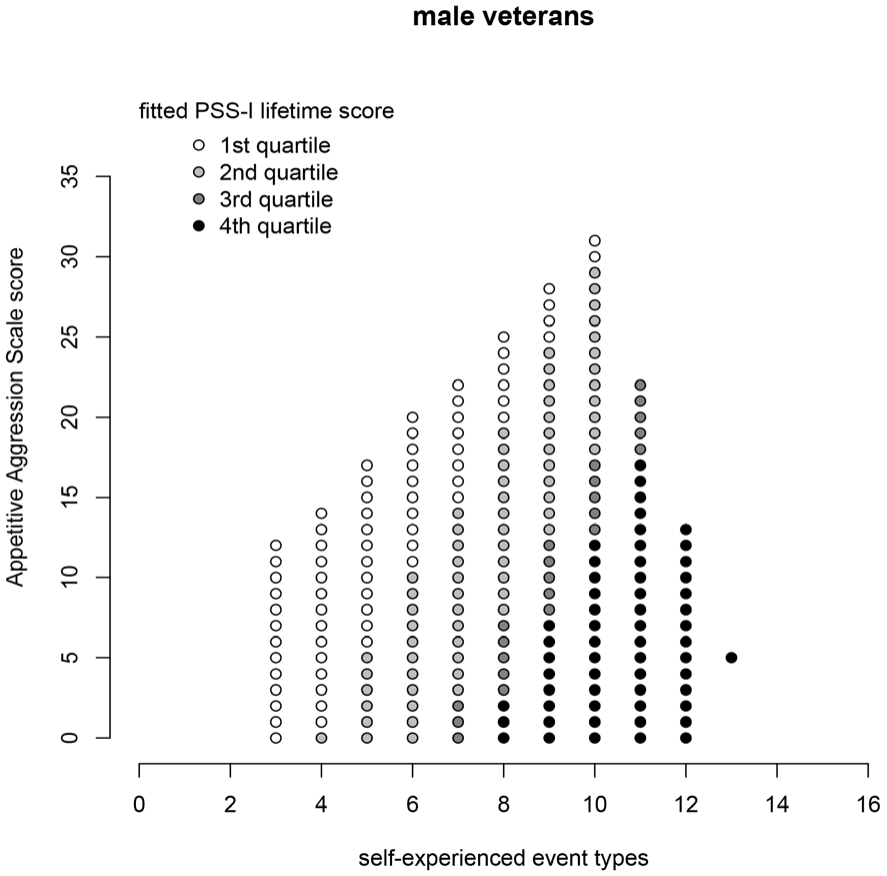
Fitted PSS-I sum scores in the group of male veterans. The grey scale indicates the predicted symptom severity. Only those PSS-I values are plotted that lie in the convex hulls of the observed predictor values in both groups.

Figure 1 illustrates the interplay between the two predictors self-experienced traumatic event types an appetitive aggression score on the PSS-I score using the example of lifetime PTSD. The fitted PSS-I sum score values that lie in the convex hulls from the observed predictor values in the data set of the veterans are displayed. Grey scale indicates the predicted symptom severity depending on the number of self experienced traumatic event types and the appetitive violence experience.

The effect sizes for the model as well as the power were calculated using the program G*Power 3 and were sufficient (*f* ^2^ = 0.51, (1-β) = .99) [40]. Thus, the sample size was large enough to prove a stable result. Moreover, there was no significant multi-colinearity in the model (maximum VIF did not exceed 1.010). The residuals also did not differ significantly from normal distribution (Kolmogorov-Smirnov *Z = *1.05, *p = *.218). There was also no severe influence of outliers in the proposed model (maximum Cook's *d* = .492).

## Discussion

The aim of the present study was to investigate potential benefits from appetitive aggression on long-term public health, especially PTSD in a sample of German World War II veterans. In agreement with our previous results [26], [27] ex-combatants who reported a higher appetitive aggression experienced less PTSD symptoms. This result is striking from two perspectives: On the one hand the effect on the current PTSD symptom severity is still present, even six decades after the end of the war. On the other hand, the resilience against trauma symptoms in those who experienced appetitive aggression during the time when they committed atrocities was present throughout the whole lifespan, as it also had an impact on the lifetime PTSD. Furthermore, all participants rated war-related events as most disturbing. This result is in line with previous findings that war-related traumatic events can cause severe and longer lasting PTSD symptoms potentially more so than non war-related traumas [41], [42]. It thus underlines once more the massive impact wars do have on mental health. Current epidmiological studies in Germany have shown that elevated PTSD rates still affect those who were directly or indirectly involved in World War II [43].

### In the “Killer-Mode”

The results from this study present further empirical evidence that the feeling of appetitive aggression is well-known to most combatants. Anecdotal evidence for a ‘lust’ for manhunt in soldiers has been reported previously, but it has not been studied systematically (e.g. [44], [45], [46]). In Holmes [47] this appetitive arousal is described as “the excitement was just fantastic, the exhilaration after all the years of training, the tremendous feeling of lift, of excitement, of exhilaration, it was like the first time you go deer hunting”. A recent publication by Neitzel and Welzer [48] that published wiretapping protocols of the ally has also documented examples of appetitive aggression. Even if it's frowned upon to talk about the lust to kill, it is a common phenomenon among soldiers and a biological predisposition of men, and not a consequence of psychopathological processes. Reports for example on Nazi leaders [49] have revealed that most people may be turned to become cruel given corresponding circumstances. Given that aggression may be perceived as appetitive among perpetrators, appropriate therapeutic interventions for the reintegration of former combatants into society, in addition to the issue of traumatisation, must in particular address a potential appeal to performing violent acts. Increasing scientific evidence requires accepting this facet of human behaviour, even though it contradicts common moral believes and values. Aggression researchers in particular have to remember that their conclusions can only be justified within the bounds of their empirical research, as views on aggressive behavior are often susceptible to moral beliefs and ideologies [50], [51]. As pointed out by Ferguson and Beaver [52], who provide a comprehensive overview on the adaptive potential of certain forms of aggression, it is naïve to assume that aggressive behavior is always pathological. The amount of appetitive aggression among former combatants that we found in our recent studies suggests that not only can moderate forms of aggressive behavior be adaptive [53], [54] but that even severe forms can be advantageous in violent environments.

### Traumatization through active killing?

Some investigators have proposed that the active involvement in killings increases the risk for trauma-related suffering. For example Breslau and Davis [55] found a 42% higher probability for PTSD in those Veterans that reported an active involvement in killings. However a greater involvement in combat is usually also related to a greater exposure to traumatic stress. Moreover, many studies demonstrated an onset of PTSD in war veterans years after the home-coming [41], [56]. In fact, symptoms of PTSD generally occur for the first time after the return from the front line. We thus suggest that there is an altered perception of violence cues during armed conflicts in combatants [20]. Our results do not contradict the concept of perpetration induced traumatic stress (PITS) introduced by MacNair [57] but we propose to refine this concept whereby two forces act on the traumatic stress: the cumulative exposure to traumatic stressors enhances and the perception of these experiences as appetitive reduces the likelihood for an extension of the associative fear or trauma network and with it mental illness. Future studies have to investigate which factors determine whether violence cues are integrated into the associative trauma network [19], [58] and thus foster PTSD symptoms or are integrated into the hunt network [20] that prevents excessive fear but increases appetitive aggression. We presume that guilt and social rejection after returning home are crucial for the re-evaluation of violent acts, shifting of violence cues from the hunt network to the fear-network. The design of successful prevention and reintegration programs requires that the potential for appetitive aggression is taken into account and where necessary properly modified.

### Limitations

The recruitment of subjects was voluntary and thus may not be representative. Moreover, PTSD is a risk factor for increased mortality [59], [60]. Consequently it is possible that a significant proportion of veterans who were severely impaired by PTSD had died six decades after the end of war. The devastating impact of war on mental health would therefore not fully appear in the present data. Moreover, veterans who committed serious atrocities may still fear condemnation of their behaviour and thus those would not have participated. Despite the reasons to assume that the variance of both measures, the one of trauma-symptoms and the one for appetitive aggression was limited by the possibilities for recruitment the relationships were found to be siginificant, i.e., effects may be even more impressive than reported here.

### Conclusion

The aim of the present study was to investigate the effects of appetitive aggression in male World War II veterans on their mental health status, especially on the risk for trauma-related symptoms during life span. Being in the killer mode during perpetration of violence constitutes a resilience factor for later development of trauma spectrum disorders. Moral-free scientific examination of appetitive aggression and the acceptance of the findings in public seem pre-requisites for the development of adequate treatment and rehabilitation programs for successful reintegration of former perpetrators, combatants or civil offenders alike, into society.
